# Proceedings of the Dental Convention

**Published:** 1856-12

**Authors:** 


### PROCEEDINGS OF THE DENTAL CONVENTION.

We have heard a number of complaints from members, in regard to the unfaithfulness with which their remarks are represented in the report of the proceedings. We have been also requested to make corrections in some cases. We have only to say that we went to the Convention prepared to employ our own reporter, or to act in concert with the editors of any, or all of the other journals, in obtaining an accurate report. Had this been carried out, we would have held ourselves *strictly* accountable for a faithful report. But the Convention took the responsibility of finding its own reporter, and furnishing the journals with an *official* report. Of course we acquiesced, both because it would have been impolite to do otherwise, and because we could save *money* by the transaction.

Well, the Convention, to relieve the editors of all responsibility, or possibly to put it beyond their power to misrepresent, furnished the proceedings in stereotype. And such had been the delay, that the only way we could get the "Register" out according to date, was to use the Convention's plates just as they came.

Now, gentlemen, if you are misrepresented, you have the consolation to know that you have misrepresented yourselves. We published your own official report, though it is full of typographical errors; and, while it misrepresents some of you by making your speeches shorter and *worse* than they were, it does equal or greater injustice to others, by representing theirs as lengthier and *better* than they really were. The former can, at the next meeting, make an *advance* on the reputation acquired by the published reports of the late meeting; while the latter must, by gigantic efforts, sustain their present acquired reputations, or be regarded as all birds are which fly high and light low. We do not intend by these remarks to reflect on the *reporter*. We regard his reputation as a fixed fact.

The probability is that some of the speeches improved more than others during the period of incubation, in which the committee sat on them. It is perhaps perfectly natural that *some* of them should. They all had *time* to grow; and it is not unnatural that those indigenous to the soil should make the most rapid progress.

In conclusion, we would say to the Convention, We are very thankful for the report—would be very thankful for another next year, but, as it will be cheaper for you, let us have it in "copy"—not in "plate." And to those who feel aggrieved at an unfaithful representation of their views, we would say, Write out your views in the shape of communications for the "Register," and we will publish them, and all will be right.

